# Coccidioidomycosis in Brazil: Historical Challenges of a Neglected Disease

**DOI:** 10.3390/jof7020085

**Published:** 2021-01-27

**Authors:** Rossana Cordeiro, Santiago Moura, Débora Castelo-Branco, Marcos Fábio Rocha, Reginaldo Lima-Neto, José Júlio Sidrim

**Affiliations:** 1 Department of Pathology, Faculty of Medicine, Federal University of Ceará, Fortaleza 60430-270, Brazil; santiago464@gmail.com (S.M.); mfgrocha@gmail.com (M.F.R.); sidrim@ufc.br (J.J.S.); 2 Postgraduate Program in Veterinary Sciences, School of Veterinary Medicine, Ceará State University, Fortaleza 60740-000, Brazil; 3 Center of Medical Sciences, Department of Tropical Medicine, Federal University of Pernambuco (UFPE), Recife-PE 50740-600, Brazil; goncalves_reginaldo@hotmail.com

**Keywords:** *Coccidioides*, coccidioidomycosis, neglected tropical diseases, fungal infection, northeastern Brazil

## Abstract

Coccidioidomycosis is a deep-seated fungal infection that occurs exclusively in semiarid areas in the Americas. In Brazil, coccidioidomycosis occurs exclusively in rural areas in the northeast region and affects counties that are hit by recurrent droughts, poverty and economic stagnation. Since 1978, approximately 136 cases of the disease have been reported in Brazil, according to scientific publications. However, a lack of governmental epidemiological data as well as a similarity to tuberculosis have led scientists and experts to assume that a greater number of cases occur in the country, which are not diagnosed and/or reported. In this review, general characteristics of coccidioidomycosis are presented, followed by a description of the main clinical and epidemiological data of cases in Brazil. The purpose of this article is to discuss the inclusion of coccidioidomycosis in the list of neglected tropical diseases. We believe that the adoption of coccidioidomycosis as a neglected tropical disease will enable the creation of an effective epidemiological surveillance system and the development of feasible public health solutions for its control in vulnerable populations.

## 1. Coccidioidomycosis: General Characteristics 

### 1.1. Occurrence

Coccidioidomycosis (CM) is a fungal infection endemic within arid and semiarid areas of the Western Hemisphere located between 40° N and 40° S latitudes. CM is caused by the two sibling species *Coccidioides immitis* and *C. posadasii*, which present subtle phenotypic differences, such as conidium size [1] and thermotolerance [2], but cause disease with similar symptomatology. A distinctive parameter between the two pathogens is their geographical distribution: while *C. immitis* occurs in southern California and, recently, in eastern Washington [3], *C. posadasii* occurs mainly in Nevada, Arizona, Texas, Utah, and Latin America [3,4].

According to the epidemiological data available, the US has the highest incidence rates of the disease among endemic countries [5], and the states of California and Arizona have the highest numbers of cases [6]. The Center for Disease Control and Prevention (CDC) Morbidity and Mortality Weekly Report [7] data showed a considerable increase in the number of CM cases in the US. Overall, the report estimated that the incidence of CM increased from 5.3 per 100,000 inhabitants in 1998 to 42.6 in 2011. However, the current incidence of the disease remains unknown in the US [5].

CM cases outside the US have also been reported. The second country in number of CM cases is Mexico, where disease prevalence is comparable to that of endemic regions in the US [8]. Mexican states located close to the US border show the highest rates of clinical cases as well as the highest indexes of cutaneous reactivity to *Coccidioides* antigens [9]. Recent studies showed that skin test surveys with *Coccidioides* antigens revealed up to 64% positive reactions in the Mexican population [10]. In addition to Mexico, CM has been reported in several countries in Latin America: Guatemala, Honduras, Colombia, Venezuela, Brazil, Paraguay, Bolivia, and Argentina. As CM is a non-notifiable disease in these countries, its real incidence in Latin America is also unknown; this hampers an understanding of the broader epidemiological picture of the disease in the Americas [5,8].

In addition to humans, CM has also been reported in nonhuman primates as well as in dogs, equine, camelids, and even in bats [11,12,13,14].

### 1.2. Mycological Features

Infection in both humans and nonhumans begins with the inhalation of aerosolized arthroconidia released by the mycelial phase (infective form) after soil disturbance. Inhaled arthroconidia may escape from phagocytosis and form a spherule (parasitic form), as a result of intricate genetic and morphological shifts. Spherules internally develop endospores, as a consequence of mitotic nuclear division, and then endospores are released after cell wall rupture. Each endospore is likely to form another spherule, resulting in an exponential increase in *Coccidioides* spp. parasitic structures. The cycle continues unless endospores return to the soil, where they undergo morphological shifts and convert to their mycelial saprobic stage [4,15].

Although *Coccidioides* species are well-recognized soil inhabitants, their recovery from natural sources is a difficult task. *Coccidioides* spp. have been isolated from alkaline soil in endemic areas [16,17,18,19,20]. Moreover, molecular detection in soil samples has also been reported [21,22,23]. A recent hypothesis presents *Coccidioides* spp. as endozoans of small mammals, instead of primary soil fungal species [24]. *Coccidioides* may live as spherules in granulomas without causing apparent disease in these mammals. When the host dies, spherules are then released from granulomas and convert into hyphae. Using dead animals as growth substrate, hyphae produce arthroconidia that may be inhaled by other small mammals, allowing the fungal life cycle to be sustained [24].

### 1.3. Clinical Aspects

CM clinical manifestations are highly variable. It is estimated that 60% of patients present asymptomatic infection that can be detected only by serological or skin tests.

Primary respiratory signs and symptoms of CM infection may be indistinguishable from common bacterial pneumonia and commonly involve cough, dyspnea, thoracic pain, fever, arthralgia, myalgia, and fatigue, occurring up to three weeks after arthoconidia inhalation [25,26,27]. Radiographic images may show lobar or segmental consolidation, multifocal consolidation, and nodules. Moreover, adenopathy and pleural effusion are also seen [28]. In endemic regions, coccidioidal pneumonia may represent up to 29% of community-acquired pneumonia [29,30]. Some patients may present erythema nodosum or multiform erythema, which are considered favorable prognostic markers [25]. Spontaneous regression of primary respiratory infection is also reported, even without antifungal therapy [26,31]. Chronic disease is assumed when clinical symptoms last beyond six weeks [32]. Alterations in imaging include pulmonary infiltrates, particularly in the apical or subapical regions [32], residual nodule, chronic cavity, persistent pneumonia with or without adenopathy, and pleural effusion [28].

Extrapulmonary disease develops in fewer than 5% of immunocompetent patients and may involve different organs and tissues [33]. Skin is the most frequently related focus of dissemination, while the central nervous system is the most severe one [33]. Meningitis is the most common clinical presentation of central nervous system disease and, without appropriate therapy, has high mortality rates. In addition, the musculoskeletal system, lymph nodes, and pericardium have also been reported as sites of dissemination [34,35]. Disseminated CM rates are higher in individuals with immunosuppression induced by previous disease or drugs as well as during pregnancy [36]. Ethnicity seems to play a role in the risk of disease dissemination; African-American descendants are about twofold more likely to progress from primary pulmonary to extrapulmonary disease [36]. When not correctly diagnosed and treated, disseminated CM may be fatal [31,36]. Therefore, accurate and early diagnosis is essential for proper patient management.

### 1.4. Diagnosis

As the clinical signs and symptoms described above are not pathognomonic, a well-performed anamnesis, consisting of a history of living or travelling to endemic regions, is extremely important for early clinical suspicion of this infection [26,31]. Although radiographic features may help to diagnose pneumonia, laboratory tests are indispensable for CM diagnosis. This is particularly true for Latin America, where tuberculosis is highly endemic and may mimic chronic CM [37,38,39].

The gold standard for CM diagnosis is direct visualization of spherules or culture isolation of the pathogen from clinical specimens [26]. The most common clinical specimens for research of *Coccidioides* spp. are those from respiratory secretions, such as sputum, bronchoalveolar lavage, and endobronchial and transbronchial biopsies. However, the fungus may also be isolated from other sites, such as urine, blood, bone marrow, skin biopsies, and cerebrospinal fluid [40,41]. Microscopic visualization of the specimens shows round spherules (10–100 µm), with a thick birefringent wall, at different stages of development. Internal or released endospores (2–5 µm) may also be observed [41]. Culture isolation of *Coccidioides* species requires biological safety level three facilities, which are not available in most clinical laboratories in Latin American countries [40].

Serological tests based on detection of immunoglobulin M (IgM) and immunoglobulin G (IgG) antibodies are also performed for CM diagnosis [6,25]. Tests can be performed in many clinical specimens, such as serum, pleural fluid, peritoneal fluid, cerebrospinal fluid, and synovial fluid [42]. Currently, detection of humoral response in CM is achieved by gel immunodiffusion, enzyme immunoassay, and lateral flow. Complement fixation and latex agglutination tests are performed less frequently [41,43]. Commercial kits are available for antibody (Immy Immuno Mycologics, Norman, OK, USA; Miravista Diagnostics, Indianapolis, IN, USA; Meridian Bioscience, Cincinnati, OH, USA) and antigen (Miravista Diagnostics, USA; Associates of Cape Cod, Inc., East Falmouth, MA, USA) detection. *Coccidioides* antigens can be detected in urine, EDTA-treated serum, cerebrospinal fluid, bronchoalveolar lavage, and other biological fluids [40].

Molecular techniques based on polymerase chain reaction (PCR) and DNA hybridization may also be useful for the diagnosis of infections caused by *Coccidioides* spp., once it enables the detection of fungal DNA in contaminated samples and clinical samples that yielded negative cultures. In addition, molecular diagnosis eliminates the risk of handling infecting filamentous cultures of the pathogen [40,44,45]. The following molecular targets showed promising results for the identification of *Coccidioides*: ITS (internal transcribed spacer) region of ribosomal DNA [46,47]; antigen-2/proline-rich antigen (*Ag2/PRA*) [45]; *Coccidioides* specific-antigen (*CSA*) [48]; and sequence of a genus-specific transposon [49]. Furthermore, matrix-assisted laser desorption/ionization time-of-flight mass spectrometry (MALDI-TOF MS) is a technology that has been used to identify yeasts, molds, and thermally dimorphic fungi, including *Coccidioides* spp. [50,51].

### 1.5. Treatment

Most cases of CM are self-limited, not requiring therapeutic intervention. In cases of mild forms of the disease, patients can receive clinical and radiological follow-up in an outpatient regimen. In severe clinical conditions, chronic and disseminated diseases require specific drug approach and/or surgery. Amphotericin B is often used in patients with respiratory failure and other severe manifestations. Since the 1980s, azole derivatives have emerged as an important therapeutic arsenal. Fluconazole and itraconazole are used in sequential or combined treatment, especially in chronic forms of the disease. The duration of therapy with azoles often varies from months to years, and for some patients, life-long suppressive therapy may be necessary in order to avoid relapses [31]. Few clinical data are available for modern azole drugs. Voriconazole and posaconazole have been claimed as successful drugs in patients who failed to respond to other azoles [52]. The cost of illness of CM in the US is high; for instance, in 2017, 7466 new CM cases were reported in California, which represented a total cost of about US $700,000 to health service providers [53]. There is no information available on the cost of CM treatment in other endemic countries.

## 2. Coccidioidomycosis in Brazil

### 2.1. Background: A Historical Overview

The first case of CM reported in Brazil occurred in 1978 [54], in a 28-year-old male patient from Pirapiranga (10°41′02″ S–37°51′54″ W), a city located in the semi-arid area of Bahia State. The patient presented respiratory symptoms, including persistent dry cough and hemoptysis; chest images showed heterogeneous opacities and cavitation in the apical segment of the left lower pulmonary lobe. The patient was improperly treated for tuberculosis, but after lobectomy, histopathological study revealed round spherules suggestive of *Coccidioides* spp. [55]. The second case of CM in Brazil was described one year later by Vianna et al. [54], in a 35-year-old male patient from Floriano, Piauí State (6°46′01″ S–43°01′22″ W), who complained of epigastric pain, without any other symptoms. X-ray imaging showed multiple pulmonary nodules, and lung biopsies revealed spherules suggestive of *Coccidioides* spp. In addition, the patient showed a positive reaction to a coccidioidin skin test. Ten years later, CM was detected again in semi-arid Brazil. A 74-year-old male patient from Jaguaribara, in Ceará State (5°39′29″ S–38°37′12″ W), who had been suffering from progressive dysphonia in the last six months without pulmonary symptoms, had a presumptive diagnosis of carcinoma, but histology showed *Coccidioides* spherules [56].

The international scientific community turned its attention to CM in Brazil after an outbreak occurred in Oeiras, Piauí State (07°01′30″ S–42°07′51″ W). In August 1991, three individuals and eight of their dogs who went on an armadillo hunt developed severe pneumonia; three dogs died a few days later. Mycological diagnosis was achieved after direct examination and culture of sputum samples. Inoculation of the same sputum samples in mice assured in vivo positive results for *Coccidioides* spp.; patients also showed positivity to spherulin skin tests [18]. Three years later, another outbreak took place in Aiuaba, Ceará State (6°34′26″ S–40°07′26″ W), when four men and two dogs presented acute pulmonary disease after armadillo hunting [57].

**Figure 1:**
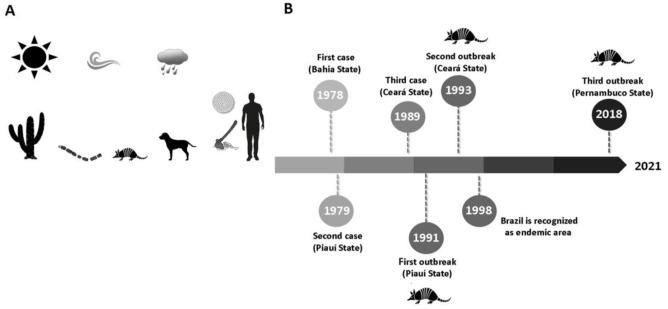
Relevant aspects of coccidioidomycosis in Brazil. (**A**) Epidemiological cycle of the disease has strong association with armadillo hunting, traditionally performed by men and assisted by dogs. The disease is limited to the semi-arid region of northeastern Brazil, an area characterized by scarce and irregular rainfall, as well as Caatinga biome. (**B**) Timeline of major events related to coccidioidomycosis in Brazil.

In 1998, Brazil was included in the international list of countries with endemic areas of the disease [58], with zones limited to the semi-arid areas of the States of Maranhão, Piauí, and Bahia. The most recent area of occurrence of CM in Brazil is the city of Serra Talhada, in Pernambuco State (7°59′09″ S–38°17′45″ W), where four individuals presented acute pneumonia after armadillo hunting [58,59,60] (Figure 1).

### 2.2. Occurrence and Epidemiological Aspects: The Disease of Armadillo Hunters

**Figure 2:**
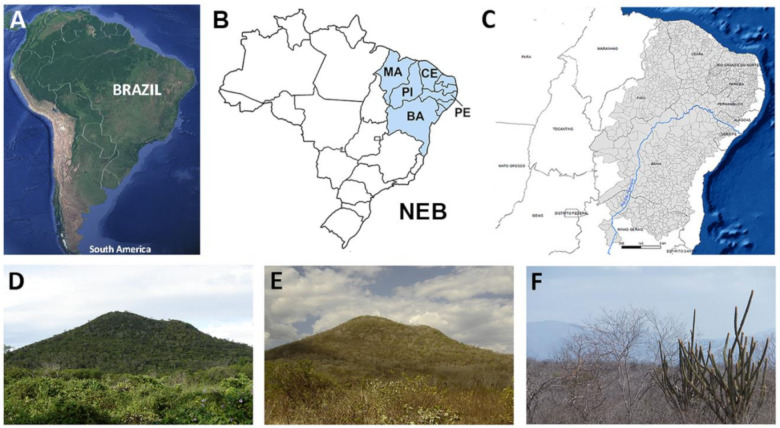
Geographic and biogeographic information of Brazil. South America map showing location of Brazil (**A**). Map depicting Brazil localization. Northeastern Brazil (NEB) is shown in blue (**B**). Coccididoiomycosis occurs in the States of Maranhão (MA), Piauí (PI), Ceará (CE), Pernambuco (PE), and Bahia (BA). All States are located in the semi-arid area (grey) of NEB (**C**). Caatinga vegetation in Ceará State during rainy (**D**) and dry seasons (**E**). Detail of the Caatinga vegetation during the dry season showing xerophytic plants and deciduous trees and shrubs (**F**).

In Brazil, CM occurs exclusively in the northeast region, where approximately 136 cases of the disease have been reported since 1978: 94 cases in the state of Piauí [61]; six cases in the state of Maranhão [18,61,62]; 30 cases in the state of Ceará [63,64,65,66,67,68]; two cases in the state of Bahia [55,68]; four cases in the state of Pernambuco [59,60] (Figure 2).

The cases of CM reported in Brazil share several clinical and epidemiological characteristics: all patients were from the same biogeographic origin (semi-arid region), were of the same gender (male), had ages varying between 11 and 82 years, although most of them were between 25 and 45 years old, and near all participated in armadillo hunts for at least two weeks before becoming ill. All cases started between the months of September and January, during the dry season in that region. Only two patients had chronic disease, while 134 patients had acute pulmonary CM. Cutaneous manifestations were described in 32 patients and included hypersensitivity reactions, such as erythema nodosum and erythema multiforme [62]. One patient developed pericarditis without pulmonary involvement [35]. Although there are no official clinical-epidemiological data, the scientific literature indicates that only two cases of acute pulmonary disease evolved to death, despite antifungal treatment [63,69]. Most patients were treated with deoxycolate amphotericin B and oral fluconazole; a minority of patients were treated with only one of these drugs alone. Liposomal amphotericin B is not available in Brazilian public hospitals. All patients were treated at the governmental hospital network of the Brazilian Unified Healthcare System, abbreviated SUS.

The role of *Dasypus novemcinctus* armadillos (Cingulata order, Dasypodidae family) in the ecology of *C. posadasii* in Brazil was initially studied by Eulalio et al. [70], who described naturally infected animals in Piauí State. Since then, armadillos have been determined to figure as sentinels for CM in Brazil. As mentioned above, the majority of CM cases in Brazil have been linked to armadillo hunting [18,57,59,64,65,66,67], a cultural practice performed exclusively by men. Armadillos are mammals with terrestrial habits and can dig deep burrows, which makes them vulnerable to arthroconidia exposure and infection. *C. posadasii* strains were obtained after direct cultivation and/or animal inoculation of soil samples collected in or nearby armadillo burrows in Ceará State [17] and Piauí State [18]. Additionally, de Macedo et al. [22] detected *C. posadasii*-related DNA in soil samples from armadillo burrows in Piauí State.

The physical and chemical characteristics of the soils where *C. posadasii* can be found in Brazil are not known in detail. Only a single study details the granulometric and mineralogic properties of positive soil samples for *C. posadasii* [17]. Authors referred to the naturally infected soils as Luvisol type, with pH 6.5 and a high sandy texture content (52%); technical research has revealed that the salinity of semi-arid area soils from Ceará State ranges from <1% to up to 40% and the soil pH ranges from 4.1–8.6. Further studies on the environmental characteristics of CM endemic areas in Brazil may allow better correlation with the data already described for the US.

### 2.3. Epidemiological Surveys Based on Unofficial Data: How Reliable Are These Studies?

In Brazil, CM is considered a non-notifiable disease, which compromises understanding its impact in the country and hinders the establishment of surveillance and control measures. Failures in recording epidemiological data are common, and consequently, information on the disease is incomplete. As a consequence, data provided by the Brazilian government show divergences from scientific publications, which may indicate that the former probably includes other diseases, such as paracoccidioidomycosis.

For instance, the search for deaths caused by CM between 1996 and 2017 in the Notifiable Diseases Information System from SUS revealed 48 events, distributed as follows: five in the north region, 22 in the northeast region, nine in the southeast region, 11 in the south region, and one in the midwest region. However, these governmental data are incoherent and unreliable, since the disease occurs exclusively in the northeast region. In turn, data obtained by the Department of Information Technology of the Brazilian Unified Health System showed that 829 cases of CM occurred in the country in 2011, a remarkably high number of cases, considering the scientific notification in that period. Data provided by the Hospital Admissions Information System from SUS show 400 cases of CM per year between 2000 and 2007, most of which were reported in the south and southeast regions (MS, 2011). Once again, government data seem to mistakenly report other infections as CM, since the south and southeast regions of Brazil do not present the adequate biological conditions for the occurrence of the disease.

Among the most important strategies for epidemiological investigation in populations exposed to fungi is intradermal testing. This approach, which uses spherulin or coccidioidin, structural antigens obtained from the parasitic and saprophytic phases of *Coccidioides* spp., respectively, has been used in the US since the 1940s. So far, only two tests of this nature have been performed in Brazil. In 1991, Wanke et al. [18] performed the intradermal spherulin test on 20 individuals associated with the first CM outbreak, in Oeiras, Piauí, and they found reactivity in only one of them. In 1993, Diógenes et al. [71] carried out an epidemiological survey with spherulin in 87 residents of Jaguaribara, Ceará State, where the third case of CM was registered. The study demonstrated reactivity in 23 individuals, thus suggesting the occurrence of CM in that region.

This scenario suggests that, although the disease has been occurring for over three decades in northeastern Brazil, and despite the efforts made by local research groups, government authorities responsible for epidemiological surveillance continue to ignore the existence of the disease in the region.

### 2.4. Northeastern Brazil at a Glance: Biogeographical Aspects, Social Characteristics, and Health Issues in a Singular Region

Northeastern Brazil (NEB) is a region located between 2.5° to 16.1° S and 34.8° to 46° W and encompasses the States of Maranhão, Piauí, Ceará, Rio Grande do Norte, Paraíba, Pernambuco, Sergipe, Alagoas, and Bahia. It corresponds to an area of approximately 1,540,000 km^2^, or about 18% of the Brazilian territory, with more than 53 million inhabitants, or almost 25% of the Brazilian population. Inside NEB there is a vast area classified as semi-arid, located between 3° to 18° S and 35° to 46° W. This semi-arid region occupies 982,563.3 km^2^, with nearly 22,600,000 inhabitants, or almost 12% of Brazilian population, distributed among 1262 municipalities. The area of NEB comprises the most populous semiarid region of the world [72].

The predominant biome in NEB is the Caatinga Phytogeographic Domain (nearly 52.5% of NEB with 844.453 km^2^), followed by the Savanna/Cerrado (29.4%), Atlantic Forest (10.7%), and Amazon (7.4%). The Caatinga occurs only in NEB, and it is formed by xerophytic plants, mostly deciduous trees and shrubs, and annual therophytic herbs [73], with more than 3300 plant species, of which over 500 species are endemic [74].

In this region, the mean rainfall is typically below 1000 mm per year, which is concentrated within a few months per year. During most of the year, rainfall is absent or negligible. Droughts are recurrent and have been reported since the 16th century. Since that time, at least 54 long-lasting droughts have been reported, accounting for 31 drought years during the 20th century and 11 drought years in the 21st century [75]. In the semi-arid region, the typical mean annual rainfall is below 800 mm, and climate studies reveal that this area presents a risk of drought above 60%. The inappropriate use of soil, based mainly on slash-and-burn practices, has contributed to intense land degradation. Many rural areas in NEB are in the process of desertification and are no longer cultivated. In addition, extensive livestock farming is practiced in many areas, intensifying soil degradation [76].

NEB also presents important social and demographic characteristics. The official population census [77] reveals that its population is formed by brown (62.5% vs. 46.8% in Brazil), black (11.9% vs. 9.4% in Brazil), white (24.7% vs. 42.7% in Brazil) and native Brazilian (0.8% vs. 1.1% in Brazil) people. The NEB population has the lowest average of schooling years (6.7 years vs. 8.0 in Brazil). In this region, 52.6% of the population has not finished primary education. Nearly 20% of the population age 25 or over living in NEB is uneducated (vs. 11.2% in Brazil). In 2016, the estimated illiteracy rate among people age 15 and over in NEB was 14.8% (vs. 7.2% in Brazil). Overall, the smallest proportion of people in Brazil with complete higher education (9.9%) is found in NEB [78].

It is believed that the rainfall irregularity and scarcity, the fragile soils, the intense land degradation and desertification, as well as the socioeconomic characteristics of its inhabitants are important factors that make NEB one of the world’s most vulnerable regions to climate change [76]. This condition, in turn, may exacerbate regional poverty and inequalities in a region historically marked by social and economic imbalances.

Official economic data show that nearly 6.4 million people living in NEB (almost 12% of its total population) have daily earnings below US $1.90 and are regarded as people living in extreme poverty by the World Bank [79]. Considering the prevalence of CM in NEB, it is clear that the disease is present in areas with economic and social vulnerabilities; in accordance with the Atlas of Human Development in Brazil, issued by the United Nations Development Programme, of the 35 municipalities where cases of CM have been reported, 16 have low a Human Development Index (HDI, 0.500–0.599), while the remaining 19 municipalities present a medium HDI (0.600–0.699) [80].

Such economic and social inequalities have made NEB’s population historically vulnerable to many infectious diseases. Local authorities face bureaucratic problems in hiring human resources, and a shortage of physicians is a recurrent problem in rural areas [81]. Moreover, structural problems in the Brazilian health system, including flaws in governance, lack of funding, and suboptimal resource allocation [81], have led to the emergence/reemergence of infectious diseases such as whooping cough, measles, congenital syphilis, and leprosy in the region [82]. In addition, many severe diseases are also endemic in NEB and cause significant health issues, including vector-borne diseases (Chagas disease, dengue fever, Zika, Chikungunya), vaccine-preventable diseases (tuberculosis, meningitis, pneumonia), water-transmissible diseases (schistosomiasis, diarrhoea), and HIV/AIDS. Of the 20 diseases recognized by the World Health Organization as neglected tropical diseases, 17 of them occur in Brazil, and 14 are endemic in NEB [83].

## 3. Neglected Tropical Diseases

Neglected tropical diseases (NTDs) are a diverse group of infectious diseases primarily associated with poverty and limited access to healthcare systems and education that affect more than one billion people in rural or peri-urban areas in low- and middle-income countries in Africa, Asia, and Latin America [84]. As they affect mainly poor people, NTDs result in loss of productivity and cost billions of dollars every year to developing economies, further aggravating poverty [84]. Therapy for such diseases includes highly toxic drugs that may potentially cause severe adverse side effects. In some cases, there is no provision of curative therapy, and treatment relies on symptom alleviation [85]. In addition, local governments have mostly failed in controlling NTDs, and some diseases are underreported in several countries [86,87].

According to the WHO [88], NTDs are those that (1) affect disproportionately populations living in poverty, resulting in high morbidity and mortality; (2) affect primarily populations living in tropical and subtropical areas; (3) may be controlled or eliminated by preventive chemotherapy, intensified case management, vector control, veterinary public health, safe water supply, sanitation and hygiene; and (4) are neglected by research. Therefore, according to these criteria, the NTD portfolio is composed of the following diseases: buruli ulcer; Chagas disease; dengue fever and chikungunya; chromoblastomycosis; dracunculiasis; echinococcosis; foodborne trematode infection; human African trypanosomiasis; leishmaniasis; leprosy; lymphatic filariasis; mycetoma; onchocerciasis; rabies; scabies; schistosomiasis; soil-transmitted helminthiases; snakebite envenoming; trachoma; and yaws [89]. Among these diseases, only dracunculasis, human African trypanosomiasis, and yaws are not reported in Brazil. Except for buruli ulcer, echinococcosis, and onchocerciasis, the remaining NTDs are endemic in NEB.

Brazil accounts for the largest number of cases of dengue fever, leprosy, schistosomiasis, Chagas disease, and leishmaniasis in the Americas, most of which occur in areas with low socioeconomic indexes, such as NEB. Approximately 10,000 NTD-related deaths are recorded in Brazil annually, and it was estimated that, in 2016, NTDs caused 475,410 disability-adjusted life-years (DALYs) in the country [90]. In Brazil, NTDs significantly contribute to the loss of health in individuals at all ages, with a higher burden among males, children under 1 year, and people age 70 and above [90].

Fungal infections are also prevalent in Brazil and are estimated to affect more than 3.8 million people [91]; in some cases, the financial impact of these infections on public health may exceed US $100,000 per patient [92]. However, among such infections, only mycetoma and chromoblastomycosis are included in the WHO NTD list [89]. Therefore, specialists have pleaded for the inclusion of other mycoses in the WHO NTD list, such as cryptococcosis, entomophthoramycosis, histoplasmosis, lacaziosis, paracoccidioidomycosis, and sporotrichosis [93,94,95,96]. According to these authors, all aforementioned diseases fulfill the WHO criteria for NTD, once they are important causes of morbidity and mortality affect primarily the poorest people living in rural areas or in areas lacking basic services, such as clean water, sewer systems, and paved roads; and are neglected by research and the pharmaceutical industry.

At the present time, the coronavirus disease 2019 (COVID-19) pandemic is a demanding health problem faced by more than 180 counties [97]. The Severe Acute Respiratory Syndrome Coronavirus 2 (SARS-CoV-2) has been a concern in Latin American countries since the first disease case was reported in Brazil in February 2020 [98]. Since then, Brazil has been recognized as one of the leading countries in number of cases of and deaths due to SARS-COV-2 infection [97]. According to the Economic Commission for Latin America and the Caribbean (ECLAC) report [99], the COVID-19 pandemic will drastically impact Latin America and the Caribbean [100], and it is expected that people living in rural areas will be the most affected. It is estimated that about 30 million people living in these areas will reach extreme poverty, which means an increase of 4.9% (from 20.3% in 2019 to 25.2% in 2020) in this population [99]. The COVID-19 pandemic will have financial and structural impacts on the health systems of affected countries, which may have repercussions on NTD control and management in the poorest regions of Latin America.

## 4. Could Coccidiodomycosis Be Considered a Neglected Disease?

A review of CM cases in Brazil suggests the disease fulfills the WHO criteria for NTD: (1) The disease occurs in rural areas of NEB, affecting mainly socially and economically vulnerable people. Our experience shows that patients often do not find adequate treatment in their local cities, being forced to travel long distances to more advanced centers in search of hospitalization. (2) Logistical difficulties and economic limitations occasionally have led to discontinuation of treatment by the patient, himself. The impact of early treatment interruption on the health and quality of life of these patients is unknown. (3) Government authorities have little control over the disease, which leads to systematic failures in prevention, treatment, detection, and reporting of cases. Misdiagnosis of CM as tuberculosis may occur, since the latter is endemic and has a high incidence in Brazil [65,101], which can worsen the clinical condition of some patients, due to lack of suitable treatment. (4) Over the decades, few financial resources have been allocated to research focused on improving diagnosis as well as the development of more effective drugs. National health authorities have failed to establish health education measures to prevent the disease, especially in remote rural areas.

On the other hand, CM has been considered a neglected disease in the US [102]. In that country, the incidence of CM has increased over the years [7], and research on this severe fungal infection is scarce [5]. Although a prophylactic vaccine for CM seems to be feasible [15,103], the pharmaceutical industry has shown little interest. However, as the production of a safe vaccine has high cost due to patents as well as animal and human tests, pharmaceutical companies are necessary for its development [102].

The recognition of CM as a neglected disease in Latin America will allow for the establishment of measures to define its prevalence in Brazil. With this information, a risk assessment and epidemiological surveillance system can be created, allowing for the establishment of specific prophylactic measures in high-risk populations as well as the development of health education strategies. Taken together, these strategies may reduce the environmental risks of acquiring CM in areas already impacted by poverty and the high incidence of other infectious diseases.

## 5. Conclusions

CM is an important underreported disease that has been occurring in Brazil for over three decades, but has been overlooked by public health authorities. The disease occurs in areas extremely vulnerable to climate change and in poor populations facing economic and social challenges. The recent economic crisis afflicting the country as well as the present COVID-19 pandemic has worsened access to health services by local populations, especially those living in rural areas. The data presented here suggest that the CM fits the definition of an NTD. The inclusion of CM in the WHO NTD list could support efforts for the prevention and control of the disease, especially in economically vulnerable populations. The Brazilian government needs to establish public policies for the prevention and control of this disease as well as to allocate a greater amount of financial resources in scientific research to expand the knowledge on the impact of CM in the country.
